# Impact of physical activity on executive functions: a moderated mediation model

**DOI:** 10.3389/fpsyg.2023.1226667

**Published:** 2024-01-04

**Authors:** Guoguo Zhao, Kaihong Sun, Jian Fu, Zhe Li, Dongbin Liu, Xin Tian, Jiehui Yang, Qiushi Zhang

**Affiliations:** ^1^Changzhou Vocational Institute of Textile and Garment, Changzhou, China; ^2^College of Physical Education, Yangzhou University, Yangzhou, China; ^3^Yangzhou Polytechnic Institute, Yangzhou, China; ^4^Guangdong Polytechnic of Environment Protection Engineering, Foshan, China; ^5^Graduate School of Physical Education, Dankook University, Yongin, Republic of Korea

**Keywords:** executive function, negative emotions, self-efficacy, physical exercise, college student

## Abstract

**Objective:**

To provide both empirical support and a theoretical framework for systematically improving and optimizing the cognitive capabilities of college students through physical activity, while considering the mediating and regulating impacts of self-efficacy and negative emotion.

**Methods:**

The study employed an overall random sampling method, examining 500 college students from five universities in Jiangsu Province using the Physical Activity Rating Scale (PARS-3), Adult Executive Function Scale (ADEXI), Positive and Negative Emotion Scale (PANAS), and General Self-Efficacy Scale (GSES).

**Results:**

The findings indicated that the average age of the participants was 18.41 ± 0.73 years, encompassing 215 male students (43%), and 185 female students (57%). Engagement in physical activity was significantly and positively correlated with executive function (*β* = 0.246, *p* < 0.01), inversely associated with negative emotion (*β* = −0.137, *p* < 0.01), and demonstrated a significant positive predictive impact on self-efficacy (*β* = 0.183, *p* < 0.01). Self-efficacy was observed to partially mediate the relationship between executive function and physical activity. In addition, negative mood was identified as playing a partial mediating and modifying role in the relationship between executive function and physical activity.

**Conclusion:**

Increasing college students’ daily physical activity participation not only benefits their executive function, self-efficacy, and confidence levels but also exerts a limited positive impact on negative mood, with the potential to regulate the intensity of negative emotion.

## Introduction

The relationship between sports and cognitive enhancement is a relatively recent yet persistent field of study. This empirical research primarily focuses on determining the optimal period for enhancing cognitive function through physical activity. College students, in a crucial stage of physical and psychological development, significantly benefit from healthy growth during this phase, thereby affecting their future development. The examination of the correlation between physical exercise and executive function gained traction in the 1970s, marked by Hillman et al. (2009) neuroimaging studies illustrating the long-term impact of exercise on the structure and function of the prefrontal cortex. Furthermore, Salthouse et al. proposed that certain executive functions, such as inhibition and working memory, serve as indicators of the natural cognitive decline associated with aging, and engaging in physical activity may help delay this decline. Chen et al. (2011) found that a 30-min session of moderate-intensity aerobic exercise positively transformed brain activation patterns related to executive function in primary school students. Similarly, Liu observed that varying doses of aerobic exercise positively impacted the executive function of college students. Consequently, researchers have recognized the positive effects of physical exercise on individuals, particularly in terms of their physical and mental well-being and behavior. However, previous studies have predominantly focused on children and the elderly (Diamond and Lee, 2011; Gawrilow et al., 2016; Benzing et al., 2018; de Greeff et al., 2018; Mora-Gonzalez et al., 2018; Yoon et al., 2018; Jirout et al., 2019; Lenze et al., 2022; Liang et al., 2022). Given that college students are in a critical period of physiological and psychological development, it is imperative to specifically investigate this aspect within this population.

People are often influenced by emotional experiences in life. Positive emotions can enhance an individual’s work efficiency, learning effectiveness, and mental health. Conversely, negative emotions are harmful to normal learning and work processes, with excessive negativity leading to conditions such as anxiety, depression, and other psychological disorders (Thorel, 2013; Liu, 2014; Jiang et al., 2016; Liu, 2021; Yang and Xiang, 2021a; Wang et al., 2023). Escalating stress in modern society exacerbates problems such as anxiety, depression, and insomnia. Researchers have taken an interest in the impact of physical exercise on emotions, with numerous empirical studies demonstrating that moderate and well-designed physical activity not only improves the physical fitness of adolescents but also boosts their cognitive abilities through neurophysiological and psychological mechanisms. Additionally, it can positively impact their emotional well-being and reduce negative states such as depression and anxiety (Blanco-Gómez, et al., 2015; Álvarez-Bueno, et al., 2017; Fu and Liu, 2018; Jiang et al., 2018; Huang, 2020; Wang et al., 2023). Motl et al. (2005) and others have observed that, with a specific duration and intensity of exercise, the elderly can mitigate negative emotions. Guszkowska (2004), through an analysis of previous studies, identified a positive effect of physical exercise on improving emotional states. Currently, emotional issues among college students are increasingly prevalent. Physical exercise is an integral part of college students’ daily lives and has a substantial impact on their emotional experiences. This study investigates the impact of physical exercise on college students’ emotional experiences and whether it can enhance their executive function by improving positive emotional experiences.

Self-efficacy, a stable psychological trait, refers to an individual’s confidence and conviction in their ability to overcome challenges and accomplish specific goals. Bandura (1987) proposes that an individual’s overall self-efficacy is shaped by their experiences of success and failure. Engaging in physical exercise can instill a sense of accomplishment, thereby bolstering self-efficacy. Moreover, physical exercise involves interpersonal communication and interaction, addressing diverse psychological needs and further reinforcing personal self-efficacy. Research by Wei Wenjing highlights the mediating role of self-efficacy and self-esteem in the relationship between physical exercise and mental health. McAuley et al. (1993) and others have observed a mutual reinforcement between self-efficacy and physical exercise behavior, where self-efficacy both initiates and sustains physical exercise behavior. Both short-term and long-term regular physical exercise have been shown to enhance self-efficacy. In addition, Zhang et al. (2017) discovered that physical exercise significantly delays the decline of executive function in the elderly, and consistent physical exercise positively influences the overall self-efficacy of older adults. Gao and Meng (2013); Gao et al. (2015) also explored the mediating role of self-efficacy between physical exercise and cognitive enhancement in older adults, revealing that physical exercise exerts both direct and indirect effects on cognitive function, with the indirect effects being more pronounced than the direct effects.

**Figure 1:**
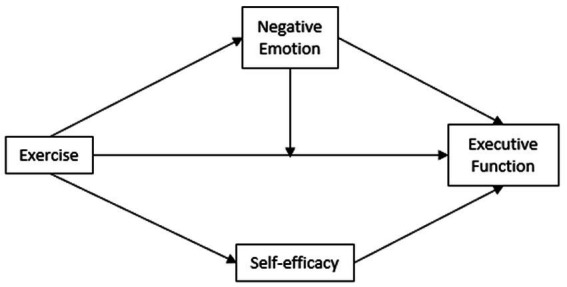
Study the hypothesis model.

Expanding on prior research, this study delves into the psychological mechanism underpinning the impact of physical exercise on the executive function of college students. This study introduces a comprehensive psychological mediation model to analyze and discuss potential intermediary factors between physical exercise and executive function. The hypothesis posits that physical exercise exerts both direct and indirect impacts on the executive function of college students. Self-efficacy and positive and negative emotions are identified as effective mediating variables that transmit these effects. Furthermore, the study aims to discern the direct and indirect consequences of physical exercise on the executive function of college students (see Figure 1). We anticipate that the findings will contribute to the theoretical framework for enhancing executive function in college students through physical exercise. Additionally, the study may offer insights into adjusting the timing and intensity of exercise to optimize executive function and foster mental resilience.

## Materials and methods

### Participants

**Figure 2:**
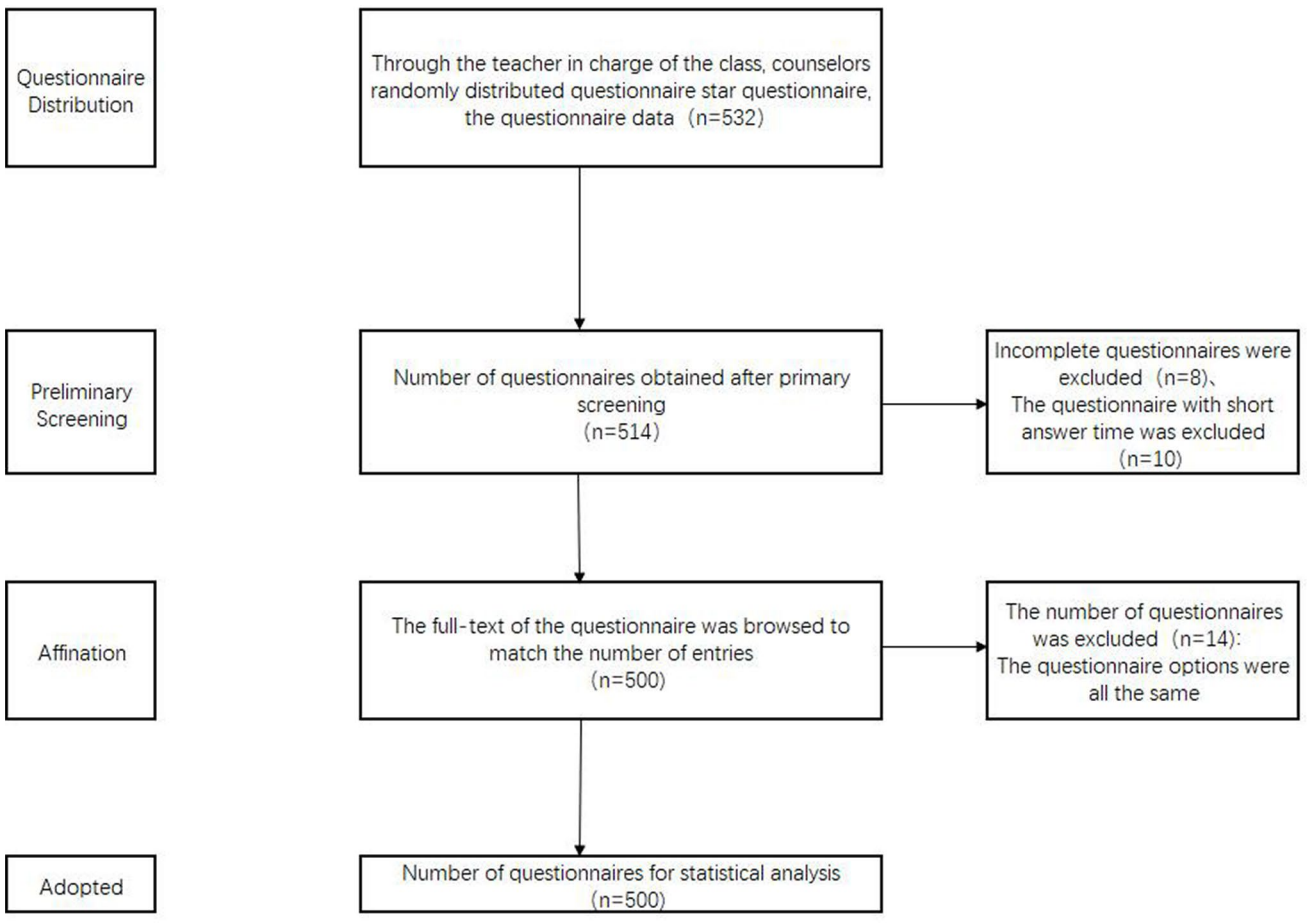
Flow chart of questionnaire data acquisition and processing.

The participants in this study were college students aged 18–23. Nonprobability sampling was used for participant selection, supplemented by additional eligible samples obtained from teachers and counselors. The questionnaire explicitly outlined the survey’s objectives and data usage, emphasizing the significance of anonymity, authenticity, and voluntary participation. A total of 532 questionnaires were distributed, and any invalid data, such as duplicate responses or excessively brief completion times, were excluded. The final analysis included 500 valid questionnaires, yielding an effective response rate of 93.98%. The process of questionnaire collection and screening is shown in Figure 2. The participants’ mean age was 18.41 ± 0.73 years, with 221 male and 279 female students. Among them, 353 were from liberal arts backgrounds, 147 were from science backgrounds, 198 were from urban areas, and 302 were from rural areas.

### Measures

#### Physical activity rating scale—3, PARA—3

This scale was devised by Japanese researcher Otake et al. (1990) to assess engagement in physical exercise. It was then translated and refined by local researchers Liang (1994). Employing a Likert 5-point scale with three items (intensity, frequency, and time). The overall score is computed by multiplying the frequency of physical activity by intensity and time. A larger overall score indicates a greater amount of exercise and a higher grade. In the context of this study, the scale demonstrated a Cronbach’s coefficient of 0.72. the scale demonstrated a McDonald’s ω coefficient of 0.87.

#### Negative emotions

The Positive and Negative Emotion Scale (PANAS), developed by Watson et al. (1988) and validated by Huang et al. (2003), was employed to assess emotions in this study. The scale comprises 10 items categorized into positive and negative emotions. A Likert 5-point scale was used, with scores ranging from 0 to 5. The intensity of negative emotions increases as the score increases. Notably, in this study, positive questions were reverse-scored to align with the negative emotion category. The Cronbach’s alpha for this scale in the study was calculated to be 0.82. the scale demonstrated a McDonald’s ω coefficient of 0.83.

#### Self-efficacy

The General Self-Efficacy Scale (GSES), developed by Schwarzer and Aristi (1997); Schwarzer et al. (1999) and subsequently refined by Wang et al. (2001), served as the measurement tool in this study. The scale comprises 10 questions, scored using a Likert 4-point system with values ranging from 1 to 4. A higher score indicates increased confidence in one’s abilities. For this investigation, the Cronbach’s coefficient for the scale was determined to be 0.89. the scale demonstrated a McDonald’s ω coefficient of 0.86.

#### Adult executive function scale

The Adult Executive Function Scale, developed by Thorell et al. (2013) and subsequently validated by Liu et al. (2022), comprises 14 items related to working memory and inhibition. Using a Likert 5-point scale, with values ranging from 1 to 5, the scale assesses executive function, where a higher score indicates better performance. In the context of this study, the Cronbach’s coefficient for the scale was determined to be 0.86. the scale demonstrated a McDonald’s ω coefficient of 0.89.

#### Process and analysis

Participants were explicitly notified that the questionnaire would be completed anonymously, treated with strict confidentiality, and its content would be solely used for scientific research purposes. The study was approved by the ethical committee of the Bio-X center at Changzhou Vocational Institute of Textile and Garment and adhered to the guidelines outlined in the Declaration of Helsinki.

Descriptive, correlation, variance, and hierarchical regression analyses of correlated variables were conducted using SPSS 27.0 (IBM Inc., Chicago, IL, United States). Additionally, the mediation roles of self-efficacy and negative mood in the relationship between exercise and executive function were investigated using AMOS 28.0 (IBM Inc., Chicago, IL, United States).

## Results

### Descriptive statistics and related analysis

To examine the impact of physical exercise, self-efficacy, and negative emotion on the executive function of college students, we conducted a correlation analysis. The summarized results are presented in Table 1. The outcomes revealed a positive correlation between self-efficacy and executive function, along with the level of physical exercise, working memory, and inhibition ability. Higher self-efficacy levels were linked to enhanced development in physical exercise, executive function, and its sub-dimensions. In addition, heightened self-efficacy was associated with a decrease in the expression of negative emotions. Physical exercise also exhibited positive correlations with self-efficacy and executive function and a negative correlation with negative emotions. These findings imply that increased engagement in physical exercise is linked to improved performance in terms of self-efficacy and executive function, as well as reduced expression of negative emotions. The correlations established in this study provide a foundation for constructing and testing the meditation model of physical exercise and executive function.

**Table 1:** Mean, standard deviation, and correlation of variables.

	M	SD	1	2	3	4	5	6
1. Self-efficacy	31.190	6.910	1					
2. Executive function	57.320	9.462	0.430^**^	1				
3. Exercise	45.670	29.285	0.183^**^	0.246^**^	1			
4. Negative emotion	44.060	21.842	−0.178^**^	−0.196^**^	−0.137^**^	1		
5. Working memory	37.050	6.020	0.422^**^	0.973^**^	0.244^**^	−0.204^**^	1	
6. Inhibiting ability	20.270	3.869	0.395^**^	0.932^**^	0.223^**^	−0.162^**^	0.822^**^	1

The significance level of ^**^is 0.01.

### Regression analysis

From Table 1, we can see that the above variables are related to each other. We need to know whether self-efficacy and negative emotion have mediating effects on physical exercise and executive function. To explore whether self-efficacy is a mediator between physical exercise and executive function, physical exercise and executive function were used as independent variables.

The correlation of physical exercise, self-efficacy, negative emotion and executive function were statistically significant (*p* < 0.01), which accorded with the test condition of intermediary effect. After centralizing the data of physical exercise amount, positive and negative emotions, self-efficacy and executive function in SPSS, we adopted the standard test procedure of mediating effect proposed by Wen et al. (2005), to investigate the mediating effect of self-efficacy and positive and negative emotions between physical exercise and executive function. Step 1 tests the total effect C of the independent variable on the dependent variable, and if significant, step 2 tests the path coefficients a of the independent variable on the intermediary variable and B and C of the independent variable on the dependent variable, if both are significant, the mediating variables have partial mediating effect, and the mediating effect value is calculated by AB/C.

From Tables 2, 4, we can see that the regression coefficient of self-efficacy has a significant correlation, indicating that it plays a part of mediating role between physical exercise and executive function, the mediating effect of self-efficacy between physical exercise and executive function accounted for 11.34% of the total effect[ab/c = (0.164 × 0.110)/0.159 ≈ 0.1134];.

**Table 2:** The mediating effect of self-efficacy between physical exercise and executive function.

Regression equation		Global FIT index		Regression coefficient	
Result variable	Predictive variables	*R* ^2^	*F*	*β*	*t*
Negative emotion	Exercise	0.023	9.543	0.159	3.089^**^
Self-efficacy	Exercise	0.024	10.089	0.164	3.176^*^
Negative emotion	Self-efficacy exercise	0.172	39.134	0.388	2.276^*^
				0.110	8.061^***^

The significance level of ^*^ is less than 0.05; The significance level of ^**^ is less than 0.01; The significance level of ^***^ is less than 0.001.

From Tables 3, 4, we can see that the regression coefficients of negative emotions are significantly correlated, which indicates that negative emotions play a part of mediating role between physical exercise and cardiac function, the mediating effect of negative emotion between physical exercise and executive function accounted for 11.89% of the total effect [ab/c = (−0.138 × −0.137)/0.159 ≈ 0.1189].

**Table 3:** The mediating effect of negative emotion between exercise and executive function.

Regression equation		Global FIT index		Regression coefficient	
Result variable	Predictive variables	*R* ^2^	F	β	t
Executive function	Exercise	0.023	9.543	0.159	3.089^**^
Negative emotion	Exercise	0.016	7.062	−0.138	−2.657^**^
Executive function	Negative emotion exercise	0.039	8.364	0.141	2.720^**^
				−0.137	−2.651^**^

The significance level of ^*^ is less than 0.05.

**Table 4:** Results of regression analysis of self-efficacy and negative emotion.

Model	Step	Standardized equations	SE	*t*
Self-efficacy	1	Executive function =0.159 × Exercise	0.032	3.089^**^
	2	Self-efficacy =0.164 × Exercise	0.034	3.176^**^
	3	Executive function =0.388 × Self-efficacy 0.110 × Exercise	0.046 0.030	8.061^***^ 2.276^*^
Negative emotion	1	Executive function =0.159 × Exercise	0.032	3.089^**^
	2	Negative emotion = −0.138 × Exercise	0.050	−2.657^**^
	3	Executive function = −0.137 × Negative emotion 0.141 × Exercise	0.034 0.032	−2.651^**^ 2.720^**^

The significance level of ^*^ is less than 0.05; The significance level of ^**^ is less than 0.01; The significance level of ^***^ is less than 0.001.

To assess the fit level of the hypothesis model, AMOS 28.0 was employed to investigate the previously posited hypotheses, H3 and H4. The mediating effect model was constructed, with the amount of physical activity as the independent variable, executive function as the dependent variable, and self-efficacy and negative mood as mediating variables. The resultant values were as follows: *x*^2^ = 31.931, *x*^2^/df = 1.521, CFI = 0.996, NFI = 0.988, RFI = 0.980, IFI = 0.996, AGFI = 0.965, and RMSEA = 0.036. In SEM, *x*^2^/df ≤ 5.00, RMSEA≤0.08, and CFI, NFI, RFI, IFI, and AGFI ≥0.900 indicate a well-fitting model, according to Wu (2009). All the indexes of the research model met the criteria for a good fit, indicating a high level of model adequacy. Figure 1 illustrates the standardized path coefficient.

**Figure 3:**
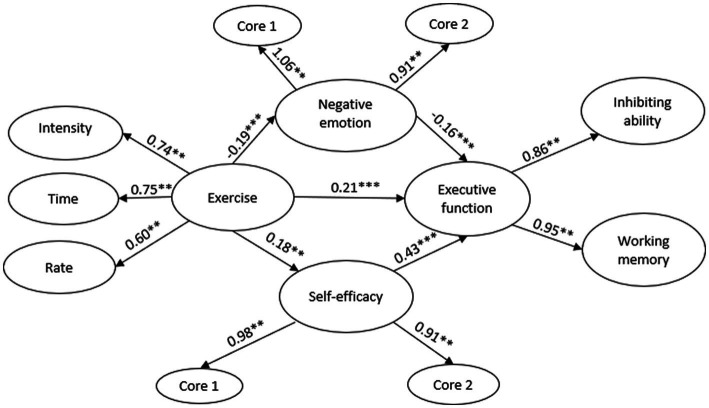
The diagram of the mediation model. ^**^is significant at the 0.05 level, ^***^is significant at the 0.001 level.

In Figure 3, the relationships between exercise and executive function (exercise executive function = 0.21, *p* < 0.001), exercise self-efficacy (exercise self-efficacy = 0.18, *p* < 0.001), exercise negative emotion (exercise negative emotion = 0.19, *p* < 0.001), and the path coefficients of negative emotion executive function (exercise executive function = 0.16, *p* < 0.001).

#### Examining the impact of negative emotions on regulation

Following the centralization of physical activity, negative emotion, self-efficacy, and executive function in SPSS, the hierarchical regression method was employed to investigate the moderating effect of negative emotion between physical activity and executive function. This was performed following the standard testing procedure for moderating effects proposed by Wen et al. (2005). In the first phase, physical activity and negative emotions were regressed to executive function, and in the second step, the dependent variable was regressed to independent variables, moderating variables, and the interaction term between independent variables and moderating variables. A moderating effect is observed if the interaction term is substantial. The findings in Table 5 indicate evidence of a regulating impact, as there were significant changes in the *R*^2^ of the interaction terms between physical activity and both positive and negative emotions (*R*^2^ = 0.005, *F* = 0.041).

**Table 5:** Results of the moderating effect of negative emotions.

Model		*β*	*SE*	*T*	*Sig*	*R^2^*	*ΔR^2^*	*F*
Executive function	(Constant)	57.935	1.242	47.735^***^	0.001			
	Exercise	0.48	0.015	3.143^**^	0.002			
	Negative emotion	−0.136	0.027	−5.115^***^	0.001	0.069	0.064	14.885
	Exercise×Negative emotion	0.006	0.001	4.430^***^	0.001	0.112	0.106	16.924

The significance level of ^**^ is less than 0.01; The significance level of ^***^ is less than 0.001.

## Discussion

According to the findings of this study, there is a closely and positively observed correlation between physical activity and college students’ levels of executive function. This aligns with previous research results (Chmura et al., 1994; Guo and Jiang, 2003; Hillman et al., 2009; Wu et al., 2022) and underscores the known benefits of physical activity for mental health and fitness. Exercise can improve both mental and physical well-being, as highlighted by Zhao et al. (2022), who reported a significant enhancement in executive function in 17 ADHD youngsters following regular exercise. Additionally, through studies involving medium and high-intensity continuous aerobic exercise studies on 48 college students, a stronger association was identified between exercise level and working memory (*r* = 0.422) compared with the association between exercise level and inhibitory function (*r* = 0.395). This posits that working memory is more strongly linked to activity levels. Physical exercise plays a crucial role in college students’ campus life as it enhances their ability to overcome setbacks and solve problems by improving their physical and psychological well-being. Consequently, engaging in regular physical exercise promotes the development of executive function among college students.

This study identified a robust positive association between self-efficacy and physical activity. Engaging in physical activity facilitates the development of relationships with friends, classmates, teachers, and other social groups, thereby fostering improved group interaction and communication. Furthermore, participating in physical activity contributes to overall physical and mental health enhancement, reducing the occurrence of depression, anxiety, and other negative emotions. Moreover, the correlation analysis revealed a significant link between physical activity and self-efficacy, indicating that physical activity can contribute to the cultivation of individual self-confidence and enhance resilience against setbacks. These findings align with the results of earlier investigations (Zhang et al., 2021; Yang et al., 2021).

The findings of this study, consistent with those of Chen et al. (2013) indicate a significant interaction between cognitive executive function and emotional state, revealing a strong negative association between negative mood and executive function. The study posits that high-level cognition can be positively influenced and regulated by emotional states, whereas cognitive function, to some extent, can control emotions. Hou (2009) also found that pleasant emotions may stimulate increased dopamine secretion in the brain, thereby enhancing personal adaptability and creativity. In addition, higher cognitive executive functioning is proposed to assist individuals in more effectively managing challenging real-life situations and reducing the generation of unpleasant emotions.

This study established the mediating role of self-efficacy between college students’ physical activity and their executive function, highlighting the interconnection between executive function, physical activity, and self-efficacy. Consistent with earlier research findings (Yang and Xiang, 2021b), this study reveals that college students’ physical activity influences executive function both directly and indirectly through self-efficacy. This underscores the significance of enhancing college students’ self-efficacy levels as a strategic approach to influencing their negative emotions through exercise. Notably, self-efficacy emerges as the factor most strongly associated with exercise behavior. Research indicates that self-efficacy can benefit from both a single vigorous physical activity (Xu and Zhou, 2018) and a sustained exercise intervention (Jo et al., 2018). These findings imply that self-efficacy plays a pivotal role as a link between physical activity and mental health. Engaging in exercise can assist college students not only in avoiding unpleasant feelings but also in elevating their self-efficacy, thereby positively impacting their mental health.

Moreover, this study reveals that the relationship between exercise and college students’ executive function is both mediated and moderated by their negative emotions. While physical activity positively predicts the executive function of college students, this impact is indirectly influenced by negative emotions (Schwarzer et al., 1997; Yin et al., 2011; Zhang et al., 2017). First, engaging in physical activity can alter a student’s attentional focus, enhance cognitive function, and elevate emotional control, all of which are interconnected with the internal formation components of negative emotions in college students. Second, college students grapple with various challenges in social, emotional, academic, and employment domains, making them susceptible to self-frustration. Those with a heightened sense of self-efficacy are better equipped to confront challenges, exhibit a more resilient will, and are less prone to adverse events.

However, there are still some deficiencies in this study. Further longitudinal experiments are needed to study the causality between these two variables. In addition, this study only considered two indicators of self-efficacy and negative emotion to improve or restrict the development of executive function. It cannot fully explain the mechanism of physical exercise. Therefore, future research needs to consider more predictive variables (such as loneliness, etc.) and combine experimental and tracking methods to further scientifically and reasonably reveal the mechanism of physical exercise in improving executive function.

## Conclusion

The moderated mediation model presented in this study elucidates how physical activity affects college students’ executive function through the mediating influences of self-efficacy and negative emotions, where the impact of physical activity on executive function is adversely affected by negative emotion. Consequently, exercise exhibits both mediating and moderating effects on executive function. It is recommended that college students pay attention to the selection of physical activity items, including intensity, time, frequency, and other factors in the participation process, ensuring not only consistent engagement but also addressing specific challenges. Regular exercise can enhance self-efficacy and reduce negative emotions, contributing to the accelerated development of executive function.

## Data availability statement

The original contributions presented in the study are included in the article/supplementary material, further inquiries can be directed to the corresponding author.

## Ethics statement

The studies involving humans were approved by the Changzhou Vocational Institute of Textile and Garment Science and Technology Division. The studies were conducted in accordance with the local legislation and institutional requirements. The participants provided their written informed consent to participate in this study.

## Author contributions

GZ wrote the manuscript. JF and KS contributed to the conception of the study and performed the manuscript. XT performed the data analyses. ZL, JY, QZ, and DL distributed the questionnaire. All authors contributed to the article and approved the submitted version.

## References

[ref1] Álvarez-Bueno C. Pesce C. Cavero-Redondo I. Sánchez-López M. Garrido-Miguel M. Martínez-Vizcaíno V. (2017). Academic achievement and physical activity: a meta-analysis. Long. Stud. Child Health Dev. 140, 1–16. doi: 10.1542/peds.2017-1498, PMID: 29175972

[ref2] Bandura A. (1987). Self-efficacy: toward a unifying theory of behavioral change. Psychol. Rev. 2, 191–215. 10.1037//0033-295x.84.2.191 847061

[ref3] Benzing V. Chang Y. K. Schmidt M. (2018). Acute physical activity enhances executive functions in children with ADHD. Sci. Rep. 8, 1–10. doi: 10.1038/s41598-018-30067-8, PMID: 30120283 PMC6098027

[ref4] Blanco-Gómez A. Ferré N. Luque V. Cardona M. Gispert-Llauradó M. Escribano J.. (2015). Being overweight or obese is associated with inhibition control in children from six to ten years of age. Acta Paediatr. 104, 619–625. doi: 10.1111/apa.12976 25690274

[ref5] Chmura J. Nazar K. Kaciuba-Uścilko H. (1994). Choice reaction time during graded exercise in relation to blood lactate and plasma catecholamine thresholds. Int. J. Sports Med. 15, 172–176. doi: 10.1055/s-2007-1021042 8063464

[ref6] Chen C. Wang Q. Liu X. Liu F. (2013). Study on the relationship between negative emotion and cognitive executive function. Adv. Mod. Biomed. 6, 1149–1152. doi: 10.13241/j.cnki.pmb.2013.06.005

[ref7] Chen A. Yin H. Wang J. Li X. Song Z. (2011). Short-term moderate-intensity aerobic exercise improves executive function in children: a magnetic resonance imagine study. China Sport Sci. 10, 35–40. doi: 10.16469/j.css.2011.10.005

[ref8] de Greeff J. W. Bosker R. J. Oosterlaan J. Visscher C. Hartman E. (2018). Effects of physical activity on executive functions, attention and academic performance in preadolescent children: a meta-analysis. J. Sci. Med. Sport 21, 501–507. doi: 10.1016/j.jsams.2017.09.595, PMID: 29054748

[ref9] Diamond A. Lee K. (2011). Interventions shown to aid executive function development in children 4 to 12 years old. Science 333, 959–964. doi: 10.1126/science.1204529, PMID: 21852486 PMC3159917

[ref10] Fu J. Liu S. (2018). Experimental research on the influence of medium-intensity physical exercise at different times. Sports Sci. 1, 114–120. doi: 10.13598/j.issn1004-4590.2018.01.017

[ref11] Gawrilow C. Stadler G. Langguth N. Naumann A. Boeck A. (2016). Physical activity, affect, and cognition in children with symptoms of ADHD. J. Atten. Disord. 20, 151–162. doi: 10.1177/1087054713493318, PMID: 23893534

[ref12] Guo B. Jiang F. (2003). Career self-efficacy theory and its application. J. Northeast Normal Univ. 5, 130–137.

[ref13] Gao X. Chai J. Meng Y. (2015). Positive effects of physical exercise on cognitive function of the elder: based on the checking of psychological intermediary model of attitude toward aging and general self-efficacy. J. Shenyang Sport Univ. 34, 7–12.

[ref14] Gao X. Meng Y. (2013). The influence of physical exercise on cognitive function: the mediate function of attitude toward aging. J. Beijing Sport Univ. 36, 93–98. doi: 10.19582/j.cnki.11-3785/g8.2013.12.018

[ref9001] Guszkowska M. (2004). Effects of exercise on anxiety, depression and mood. Psychiatr. Pol. 38, 611–620. 15518309

[ref15] Hillman C. H. Pontifex M. B. Raine L. B. Castelli D. M. Hall E. E. Kramer A. F. (2009). The effect of acute treadmill walking on cognitive control and academic achievement in preadolescent children. Neuroscience 3, 1044–1054. doi: 10.1016/j.neuroscience.2099.001.057 PMC2667807 19356688

[ref17] Hou R. (2009). The lnfluence of emotion on cognition. Psychol. Res. 2, 28–33.

[ref18] Huang J. (2020). The relationship between physical exercise and subjective well-being of vocational college students: the mediating effect of psychological elasticity. Psychology Monthly. 17, 122–123. doi: 10.19738/j.cnki.psy.2020.17.047

[ref19] Huang L. Yang T. Ji Z. (2003). A study on the applicability of the scale of positive and negative emotions in Chinese population. Chin. J. Mental Health 1, 54–56.

[ref20] Jiang Y. Zhang L. Mao Z. (2018). Physical exercise and mental health: the role of self-efficacy and emotion regulation strategies in emotion regulation. Stud. Psychol. Behav. 4, 570–576.

[ref21] Jiang X. Yuan Y. Wang E. Li H. (2016). The effects of physical exercise on positive and negative emotional experiences of college students. Chin. J. Health Psychol. 1, 126–130. doi: 10.13342/j.cnki.cjhp.2016.01.033

[ref22] Jo G. Rossow-Kimball B. Lee Y. (2018). Effects of 12-week combined exercise program on self-efficacy, physical activity level, and health related physical fitness of adults with intellectual disability. J. Exer. Rehab. 14, 175–182. doi: 10.12965/jer.1835194.597, PMID: 29740549 PMC5931151

[ref23] Jirout J. LoCasale-Crouch J. Turnbull K. Gu Y. Cubides M. Garzione S.. (2019). How lifestyle factors affect cognitive and executive function and the ability to learn in children. Nutrients 11, 1–29. doi: 10.3390/nu11081953, PMID: 31434251 PMC6723730

[ref24] Lenze E. J. Voegtle M. Miller J. P. Ances B. M. Balota D. A. Barch D.. (2022). Effects of mindfulness training and exercise on cognitive function in older adults: a randomized clinical trial. JAMA 328, 2218–2229. doi: 10.1001/jama.2022.21680, PMID: 36511926 PMC9856438

[ref25] Liang X. Li R. Wong S. H. S. Sum R. K. W. Wang P. Yang B.. (2022). (2021). The effects of exercise interventions on executive functions in children and adolescents with autism Spectrum disorder: a systematic review and meta-analysis. Sports Med. 52, 75–88. doi: 10.1007/s40279-021-01545-3, PMID: 34468951

[ref26] Liu H. Guo S. He X. Zhao X. (2022). Reliability and validity of adult executive function self-rating scale in Chinese adolescents. Chin. J. Clin. Psych. 5, 1170–1173. doi: 10.16128/J.CNKI.1005-3611.2022.05.032

[ref27] Liang D. (1994). Stress level of college students and its relationship with physical exercise. Chin. Ment. Health J. 1, 5–6.

[ref28] Liu Z. (2021). The effect of physical exercise on college students’ negative emotions ーー the mediating and regulating effect of self-efficacy and mental toughness. J. Phys. Educ. 5, 102–108. doi: 10.16237/j.cnki.cn44-1404/g8.2020.05.014

[ref29] Liu J. (2014). The positive effect of aerobic exercise on executive function of college students: an fMRI study. J. Beijing Univ. Sport 3, 77–83. doi: 10.19582/j.cnki.11-3785/g8.2014.03.013

[ref9003] McAuley E. Lox C. Duncan T. E. (1993). Long-term maintenance of exercise, self-efficacy, and physiological change in older adults. J Gerontol. 48, P218–P224. doi: 10.1093/geronj/48.4.p218 8315239

[ref30] Mora-Gonzalez J. Esteban-Cornejo I. Cadenas-Sanchez C. Migueles J. H. Molina-Garcia P. Rodriguez-Ayllon M.. (2018). Physical fitness, physical activity, and the executive function in children with overweight and obesity. J. Pediatr. 208, 50–56.e1. doi: 10.1016/j.jpeds.2018.12.028, PMID: 30902422

[ref9004] Motl R. W. Konopack J. F. McAuley E. Elavsky S. Jerome G. J. Marquez D. X. (2005). Depressive symptoms among older adults: long-term reduction after a physical activity intervention. J Behav Med. 28, 385–394. doi: 10.1007/s10865-005-9005-5 16049630

[ref16] Otake Y. Aoki M. Imamura N. Ishikawa M. Hashimoto K. Fujiyama R. (1990). Aortico-pulmonary paraganglioma: case report and Japanese review. Jpn. J. Thorac. Cardiovasc. Surg. 54, 212–6. doi: 10.1007/BF02670315 16764311

[ref31] Schwarzer R. Aristi B. (1997). Optimistic self-beliefs: assessment of general perceived self-efficacy in thirteen cultures. Word Psychol. 3, 177–190.

[ref32] Schwarzer R. Baβler J. Kwiatek P. Schroder K. Zhang J. (1997). The assessment of optimistic self-beliefs: comparison of the Chinese, Indonesian, Japanese, and Korean versions of the general self-efficacy scale. Psychology. 1, 69–88.

[ref33] Schwarzer R. Mueller J. Greenglass E. (1999). Assessment of general perceived self-efficacy on the internet: data collection in cyberspace. Anxiety Stress Copying 12, 145–161. doi: 10.1080/10615809908248327

[ref34] The Cochrane Collaboration Angevaren M. Aufdemkampe G. Verhaar H. J. J. Aleman A. Vanhees L. (2008). Physical activity and enhanced fitness to improve cognitive function in older people without known cognitive impairment. Cochrane Database Syst. Rev. 2, 1–56. doi: 10.1002/14651858.CD005381.pub2, PMID: 18425918

[ref35] Thorell L. B. Veleiro A. Siu A. F. Mohammadi H. (2013). Examining the relation between ratings of executive functioning and academic achievement: findings from a cross-cultural study. Child Neuropsychol. 19, 630–638. doi: 10.1080/09297049.2012.727792 23075095 PMC3827673

[ref36] Watson D. Clark L. A. Tellegen A. (1988). A development and validation of brief measures of positive and negative affect: the PANAS scales. J. Pers. Soc. Psychol. 54, 1063–1070. doi: 10.1037/0022-3514.54.6.1063 3397865

[ref37] Wang J. Yang Y. Yuan X. Xie T. Zhuang J. (2023). Effect of high-intensity interval training on executive function for healthy children and adolescents: a systematic review J. Chin. J. Rehabil. Theory Pract 29, 1012–1020.

[ref38] Wang C. Hu Z. Liu Y. (2001). Reliability and validity of general self-efficacy scale. Appl. Psychol. 1, 37–40.

[ref39] Wu M. (2009). Structural equation modeling: operation and application of Amos. Chongqing University Press.

[ref40] Wen Z. Hou J. Zhang L. (2005). A comparison of moderator and mediator and their applications. Acta. Psychologica. Sinica. 268–274.

[ref41] Wu J. Zhao X. Zhao W. Chi X. Ji J. Hu J. (2022). The effect of physical exercise on college students’ negative emotion: the mediating effect of self-efficacy. Chin. J. Health Psychol. 6, 930–934. doi: 10.13342/j.cnki.cjhp.2022.06.027

[ref42] Xu S. Zhou A. (2018). Effects of physical exercise and motivational videos on self-efficacy of female college students. J. Henan Inst. Educ. 4, 81–87.

[ref43] Yang J. Xiang C. (2021a). Research on the path of physical exercise promoting college students’ psychological well-being: the mediating effect of social self-efficacy. J. *Chengdu Univ. Sport* 3, 132–136. doi: 10.15942/j.jcsu.2021.03.021

[ref44] Yang Y. Wan M. Wan X. (2021). Effects of high-intensity intermittent exercise and moderate-intensity continuous aerobic exercise on executive function of college students. J. Tianjin Univ. Sport 6, 733–738. doi: 10.13297/j.cnki.issn1005-0000.2021.06.017

[ref45] Yang J. Xiang C. (2021b). A study on the path of physical exercise promoting college students’ psychological well-being: the mediating effect of social self-efficacy. J. Chengdu Sport Univ. 3, 132–136.

[ref46] YoonD. H.LeeJ. Y.SongW. (2018). (2018). Effects of resistance exercise training on cognitive function and physical performance in cognitive frailty: a randomized controlled trial. J. Nutr. Health Aging 22, 944–951. doi: 10.1007/s12603-018-1090-9, PMID: 30272098

[ref47] Yin H. Chen A. Wang J. Li X. Song Z. (2014). A follow-up study of the effects of two exercise intervention programs on the executive function of primary school students. China Sport Sci. 3, 24–28. doi: 10.16469/j.css.2014.03.001.03

[ref48] Zhang Y. Zhou C. Chen A. Yan J. Hong C. (2017). Review and prospect of the relationship between chronic exercise and cognitive function: from the perspective of international historical development. China Sport Sci. 5, 68–79. doi: 10.16469/j.css.201705007

[ref49] Zhao Y. Huang L. Li Y. (2022). The effect of physical exercise on the emotional stability of flight cadets: the chain mediating effect of perceived social support and self-efficacy. Chin. J. Health Psychol. 3, 446–451.

[ref50] Zhang J. Men K. Lei H. Lu Y. Qi Y. Li J. (2021). A cross-sectional study on the mental health of college students in Xi’an. South China J. Prevent. Med. 4, 550–552.

[ref51] Zhang L. Zha S. (2017). Influence of exercise on old People’s executive function: multi-test of intervening model. Sports Sci. 38, 94–102. doi: 10.13598/j.issn1004-4590.2017.04.014

